# Impacts of gene variants on drug effects-the foundation of genotype-guided pharmacologic therapy for long QT syndrome and short QT syndrome

**DOI:** 10.1016/j.ebiom.2024.105108

**Published:** 2024-04-22

**Authors:** Zhihan Zhao, Xiaobiao Zang, Kerun Niu, Weifeng Song, Xianqing Wang, Andreas Mügge, Assem Aweimer, Nazha Hamdani, Xiaobo Zhou, Yonghui Zhao, Ibrahim Akin, Ibrahim El-Battrawy

**Affiliations:** a Heart Center of Henan Provincial People's Hospital, Central China Fuwai Hospital, Central China Fuwai Hospital of Zhengzhou University, Zhengzhou, Henan, 450003, China; b Department of Orthopaedic, Henan Provincial People's Hospital; Zhengzhou University People's Hospital, Zhengzhou, Henan, 450003, China; c Department of Cardiology and Angiology, Bergmannsheil University Hospitals, Ruhr University of Bochum, 44789, Bochum, Germany; d Institute of Physiology, Department of Cellular and Translational Physiology, Medical Faculty and Institut für Forschung und Lehre (IFL), Molecular and Experimental Cardiology, Ruhr University Bochum, Bochum, Germany; e HCEMM-Cardiovascular Research Group, Department of Pharmacology and Pharmacotherapy, University of Budapest, Budapest, Hungary; f Department of Physiology, Cardiovascular Research Institute Maastricht University Maastricht, Maastricht, the Netherlands; g Cardiology, Angiology, Haemostaseology, and Medical Intensive Care, Medical Centre Mannheim, Medical Faculty Mannheim, Heidelberg University, Germany; h German Center for Cardiovascular Research (DZHK) Partner Site Heidelberg/Mannheim, Medical Centre Mannheim, Heidelberg University, Germany; i Key Laboratory of Medical Electrophysiology of Ministry of Education and Medical Electrophysiological Key Laboratory of Sichuan Province, Institute of Cardiovascular Research, Southwest Medical University, Luzhou, Sichuan, China

**Keywords:** Short-QT syndrome, Long-QT syndrome, Anti-arrhythmic drugs, Gene-variants, Gene variant-guided-therapy

## Abstract

The clinical significance of optimal pharmacotherapy for inherited arrhythmias such as short QT syndrome (SQTS) and long QT syndrome (LQTS) has been increasingly recognised. The advancement of gene technology has opened up new possibilities for identifying genetic variations and investigating the pathophysiological roles and mechanisms of genetic arrhythmias. Numerous variants in various genes have been proven to be causative in genetic arrhythmias. Studies have demonstrated that the effectiveness of certain drugs is specific to the patient or genotype, indicating the important role of gene-variants in drug response. This review aims to summarize the reported data on the impact of different gene-variants on drug response in SQTS and LQTS, as well as discuss the potential mechanisms by which gene-variants alter drug response. These findings may provide valuable information for future studies on the influence of gene variants on drug efficacy and the development of genotype-guided or precision treatment for these diseases.

**Search strategy and selection criteria**

We searched PubMed with the following search terms: “cardiac channelopathy and pharmacotherapy”, “long QT syndrome and pharmacotherapy”, “short QT syndrome and pharmacotherapy”, “long QT syndrome subtype and drug effect”, “short QT syndrome subtype and drug effect”, “cardiac ion channel gene mutation and drug effect”, “cardiac *hERG* channel and drug effect”, “cardiac *KCNQ1* channel and drug effect” and “*SCN5A* channel and drug effect”. We included literature from the years 2000–2023. Additionally, we searched for relevant papers published before the year 2000 through the references of some papers. Our review included clinical or experimental studies that focused the on pharmacotherapy of cardiac channelopathy or LQTS or SQTS. We also included articles that examined the impact of mutations or variants of cardiac ion channels on drug effect. Only papers published in English were included.

## Introduction

**Fig. 1:**
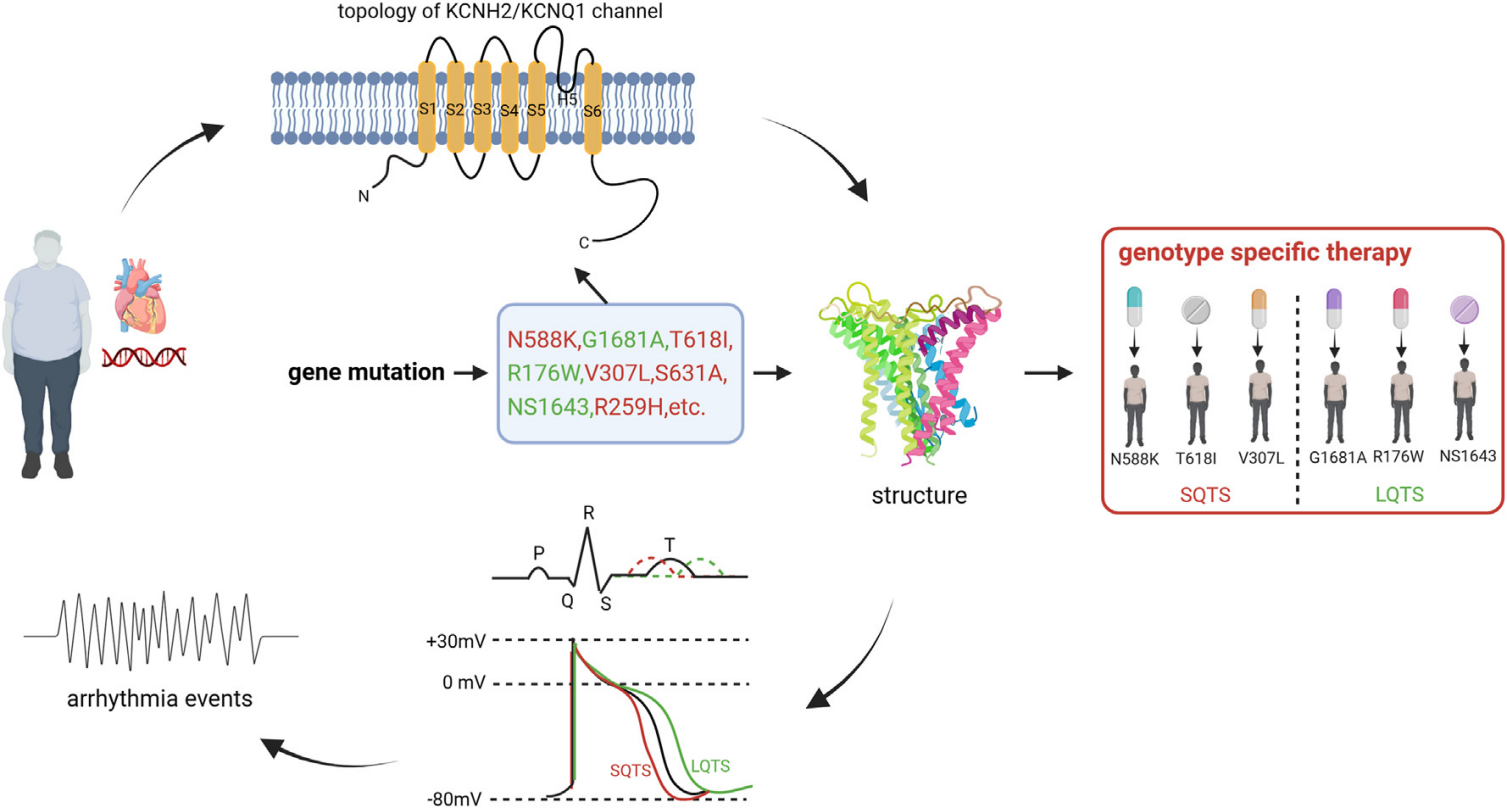
**Diagram illustrating the impact of gene mutations on ion channel function, drug effects, and clinical therapy in LQTS and SQTS.** The main genes affected in LQTS and SQTS are the human cardiac *KCNH2* and *KCNQ1* genes, which encode the hERG and Kv7.1 channels, respectively. Both channels have a pore-forming alpha subunit with 6 transmembrane domains, and a functional channel is composed of 4 alpha subunits. Gain-of-function mutations (shown in red) enhance the outward current, resulting in a shortened duration of action potentials (APD) and QT-interval on the ECG, which can cause arrhythmias. Conversely, loss-of-function mutations (shown in green) reduce the outward current, leading to prolonged APD/QT and subsequent arrhythmias. Importantly, gene mutation-induced changes in the ion channel sequence or structure can affect not only channel gating and function, but also the response to drugs. Therefore, genotype-specific therapy may be necessary for LQTS/SQTS with certain mutations. The red and green mutations shown are examples of mutations in the *KCNH2* gene.

SQTS and LQTS are congenital heart diseases that increase the risk of ventricular arrhythmia and sudden cardiac death (SCD). The prevalence of LQTS is approximately 1 in 2000 with a higher occurrence in females,^1^ while the prevalence of SQTS is estimated to be 1 in 5000 to 1 in 1000, with higher occurrence in males.^2^ Numerous variants in different genes have been identified in patients with LQTS and SQTS. The disease manifestation can vary greatly among patients with different pathogenic gene variants, ranging from asymptomatic to sudden cardiac death (SCD). This variability may be due to incomplete penetrance or the influence of non-genetic factor effects. However, the exact factors that contribute to the heterogeneity in disease presentation are not yet fully understood. In terms of treatment, different approaches, such as implantable cardioverter defibrillator (ICD) and/or pharmacotherapy, can be considered depending on patient's individual situation. Numerous studies have investigated the effects of drugs in LQTS and SQTS, and some drugs have been found to be effective. However, drug efficacy can vary greatly among patients, even among those with the same disease. Interestingly, studies have shown that gene variants can alter the effects of certain drugs.^3^^,^^4^ For example, classical *hERG* channel-blocking drugs like sotalol have been found to have no beneficial effects in SQTS patients with the N588K variant in the *hERG* channel.^5^ This variant has been shown to render the *hERG* channel resistant to some drugs.^4^^,^^6^^,^^7^ Additionally, other gene variants have been reported to impact drug efficacy in patients with genetic arrhythmias. This review will summarise the reported data on drug effects from studies conducted in patients and experimental models of LQTS and SQTS with different gene variants, and will discuss the possible mechanisms through which gene variants affect drug efficacy (see Fig. 1).

## Short QT syndrome

SQTS was first reported in 2000, when cases were presented with a shortened QT interval and an increased risk of life threatening arrhythmias.^8^ The clinical symptoms of SQTS include palpitations, syncope, dizziness, and even SCD. SCD commonly occurs in SQTS patients between the ages of 20 and 40.^9^ The ESC SQTS diagnostic criteria were updated in 2022.^10^

### Genotype-phenotype relation of SQTS

To date, several genotypes of SQTS have been identified (Table 1).^11^ SQTS1-3 are associated with dysfunction of potassium channels, including *KCNH2* (encoding Kv11.1/hERG/I_Kr_ channel, SQTS1), *KCNQ1* (encoding Kv7.1/I_Ks_ channel, SQTS2) and *KCNJ2* (encoding Kir2.1/I_K1_ channel, SQTS3) channels. SQTS4-6 are linked to dysfunction of calcium channels including *CACNA1C* (encoding the alpha-subunit of Ca^2+^ channel, SQTS4), *CACNB2* (encoding the beta-subunit of Ca^2+^ channel, SQTS5), and *CACNA2D1* (encoding the alpha2/delta1 subunit of Ca^2+^ channel, SQTS6) channels. A variant in the cardiac Cl/HCO3 exchanger AE3 gene (*SLC4A3*) was identified in patients with SQTS.^12^^,^^13^ Recently, Walsh et al. evaluated the validity of genes for SQTS and revealed that the *KCNH2* gene was classified as definitive, *KCNQ1, KCNJ2* and *SLC4A3* were classified as strong to moderate evidence. It is worth noting that, the genetic evidence for SQTS genes was derived from very few variants.^14^ The evidence for other genes, including *SCN5A, CACNA1C, CACNA2D1*, and *CACNB2,* for SQTS susceptibility is disputed.

**Table 1:** Genotype and phenotypes of short-QT syndrome (SQTS) and long-QT syndrome (LQTS).

Disease	Phenotype	Genotype	Affected current or protein	Characteristic
SQTS	SQTS1	*hERG*	IKr	Gain-of-function
	SQTS2	*KCNQ1*	IKs	Gain-of-function
	SQTS3	*KCNJ2*	IK1	Gain-of-function
	SQTS4	*CACNB2*	ICa-L	Loss-of-function
	SQTS5	*CACNA1C*	ICa-L	Loss-of-function
	SQTS6	*CACNA2D1*	ICa-L	Loss-of-function
	SQTS7	*SCN5A*	INa	Loss-of-function
	SQTS8	*SLC4A3*	H+/Cl- exchanger	Loss-of-function
LQTS	LQTS1	*KCNQ1*	IKs	Loss-of-function
	LQTS2	*hERG*	IKr	Loss-of-function
	LQTS3	*SCN5A*	INa	Gain-of-function
	LQTS4	*ANK2 (Ankyrin B)*	INa	Gain-of-function
	LQTS5	*KCNE1*	IKs	Loss-of-function
	LQTS6	*KCNE2*	IKr	Loss-of-function
	LQTS8	*CACNA1C*	ICa-L	Gain-of-function
	LQTS9	*CAV3 (Caveolin)*	INa	Gain-of-function
	LQTS10	*SCN4B*	INa	Gain-of-function
	LQTS11	*AKAP9 (A anchor protein 9)*	IKs	Loss-of-function
	LQTS12	*SNTA1 (alpha-1 syntrophin)*	INa	Gain-of-function
	LQTS13	*KCNJ5*	Ikir	Loss-of-function
	LQTS14	*CALM1 (Calmodulin1)*	Multiple currents	
	LQTS15	*CALM2 (Calmodulin2)*	Multiple currents	
	LQTS16	*CALM3 (Calmodulin3)*	Multiple currents	
	LQTS17	*TRDN (Triadin)*	Triadin	Loss-of-function

Several variants of *KCNH2*, including N588K, T618I, R1135H, E50D,^15^, ^16^, ^17^, ^18^ I560T,^19^ and S631A^20^ have been identified in SQTS1 patients. Among them, T618I and N588K are the most common, accounting for 25.9% and 18.5% respectively.^21^

*KCNQ1* variants detected in SQTS2 patients include V307L,^22^ R295H, F279I,^23^^,^^24^ F279C, V141M and A287T.^25^ D172N, M301K and E299V variants in *KCNJ2* were characterised as variants of SQTS3.^26^, ^27^, ^28^ All of these variants can cause gain-of-function of K^+^ channels and enhance K^+^ current, leading to action potential duration (APD)/QTc shortening. Some variants in calcium- and sodium-channel genes have also been detected in patients with SQTS, including *CACNA1C*-G490R,^29^ *CACNA1C*-A39V and *CACNA1C* -R1937P,^29^^,^^30^ *CACNB2*-S481L (29), *CACNA2D1*-S755T,^31^ and *SCN5A*-R689H.^25^^,^^32^^,^^33^ These variants result in the loss of function of channels and reduce calcium- or sodium-current, which leads to APD/QTc shortening. Recently, a new variant called R370H in the *SLC4A3* gene has been identified in two SQTS families^12^ and *SLC4A3* has been described as the SQT8 gene.^11^ It has been discovered that this variant causes a loss-of-function in the Cl/HCO3-exchanger (AE3) by impairing the trafficking of the exchanger protein to the cell membrane. However, the mechanism by which AE3 dysfunction leads to APD/QT shortening has not yet been clarified.

### Drug effects on short QT syndrome

Since SQTS1 is caused by gain-of-function variants in *KCNH2*, *hERG* channel blockers might be useful for treating SQTS1. However, certain *hERG*-blocking agents such as sotalol and ibutilide, were found to be ineffective in SQTS1 patients.^5^^,^^15^^,^^34^ These agents did not prolong the QTc interval or reduce the risk of ventricular tachyarrhythmias. Additionally, the *hERG* selective blocker E−4031 was found to be less effective at inhibiting the *KCNH2*–N588K variant compared to WT-channels.^35^ Subsequent studies revealed that the *KCNH2*–N588K variant impaired the inactivation of the *hERG* channel, making it insensitive to blockers. Furthermore, nifekalant,^36^ bepridil,^21^ hydroquinidine, flecainide and sotalol^37^^,^^38^ have been tested to assess their differential effects in patients with SQTS1. Bodi et al. conducted a study to examine the effects of two β-blockers on SQTS variants, *KCNH2*-N588K and *KCNQ1*-V307L, in CHO-K1 or HEK-293T cells.^39^ Their findings showed that the *KCNH2*-N588K and *KCNQ1*-V307L variants reduced the effects of carvedilol on the tail currents of *hERG* and Kv7.1 channels, respectively. However, they enhanced the effect of carvedilol on the current at end-pulse and the effect of metoprolol on both tail and end-pulse currents. Carvedilol was able to normalise the QT-interval of SQTS1, but its efficacy was slightly lower in SQTS2. These data suggest that differential effects of β-blockers can be expected in SQTS1 and SQTS2. However, there are currently no relevant clinical data available regarding the efficacy of β-blockers in SQTS patients.

In an experimental study using CHO cells with the N588K and S631A variants in *KCNH2*, they examined the effects of five anti-arrhythmia drugs: E−4031, amiodarone, quinidine, propafenone, and disopyramide, and found that the potency of these drugs in inhibiting *hERG* channel currents was differentially effected by both variants,^4^ suggesting that the variants play a role in drug effects. Another group assessed the antiarrhythmic properties of ranolazine and vernakalant in a pharmacological model of SQTS.^40^ In this study, they applied pinacidil, an activator of ATP-sensitive potassium channels, to Langendorff-perfused rabbit hearts to induce APD and QT shortening, and programmed stimulation was applied to induce arrhythmias. Both ranolazine and vernakalant have been found to successfully prolong the QT interval and suppress arrhythmias. However, it is important to note that drug-induced SQTS may differ from SQTS caused by gene variants, either in terms of pathogenesis or drug response. Therefore, the data obtained from these studies cannot be directly used to select drugs for SQTS therapy. Fortunately, thanks to the development of human induced pluripotent stem cell (hiPSC) techniques, researchers were able to establish the first cellular model of SQTS1 (*KCNH2*–N588K) using hiPSC-derived cardiomyocytes (hiPSC-CMs) in 2018.^41^ This breakthrough allowed for further investigations, which revealed that ivabradine, ajmaline, and mexiletine but not flecainide, ranolazine, or amiodarone could prolong APD and reduce arrhythmic events. Therefore, these drugs may be potential candidates for treating or preventing ventricular tachyarrhythmias in individuals with SQTS1 carrying the *KCNH2*–N588K variant.^6^^,^^7^ Other studies have shown that quinidine, disopyramide,^42^^,^^43^^,^^44^ vernakalant,^45^ but not sotalol, could prolong APD or suppress arrhythmia in hiPSC-CMs from an SQTS1-patient carrying *KCNH2*–N588K. These findings suggest that *KCNH2*–N588K may have different effects on drug responses, which could help explain why SQTS patients with *KCNH2*–N588K have varying responses to *hERG* channel blockers. Additionally, a computational framework was used to evaluate the drug effects of SQTS1, revealing that quinidine and ivabradine were more effective than ajmaline and mexiletine.^46^ It is worth noting that the computational analysis of drug effects relies on experimental data as its foundation. Moreover, the results obtained from the computational model also need to be confirmed in experimental or clinical studies.

So far, the front-line drug of choice for treating SQTS is quinidine in pharmacotherapy. Different studies have confirmed the efficacy of quinidine in SQTS1 with N588K. Computational modelling was used to assess the efficacy of quinidine in SQTS2.^47^ Quinidine was found to be effective in treating *KCNQ1*-V307L, but not in the V141M variant, indicating that quinidine may have limitations in treating SQTS with certain variants. Luo et al. discovered that high-dose amiodarone is effective for the SQTS2-V307L variant.^48^ In a patient with SQTS2 carrying *KCNQ1*-R259H, hydroquinidine was shown to be an effective medication.^38^ Additionally, the effective use of taurine-magnesium coordination compound (TMCC) in treating SQTS2 has been reported.^49^ On SQTS3, several drugs have been tested, mainly in experimental settings. These include quinidine,^50^ chloroquine,^5^^,^^51^^,^^52^ gambogic acid (GA),^53^ amiodarone,^54^ disopyramide, E−4031^55^^,^^56^ and a traditional Chinese medicine known as styrax.^57^

Theoretically, the I_Ca-L_ agonists could be effective for SQTS4-6. However, there are currently no clinically available selective I_Ca-L_ agonists for SQTS. It has been reported that quinidine might be an effective drug for SQTS patients carrying calcium channel gene variants^29^^,^^58^ El-Battrawy et al. illustrated that quinidine and amiodarone prolonged APD in a patient with SQTS overlapped with BrS carrying a variant (c.1439C > T/p. S480L) in *CACNB2* using hiPSC-CMs. Only amiodarone exhibited a significant antiarrhythmic effect. Sotalol prolonged APD in healthy cells but not in SQTS cells. Additionally, a PI3K activator prolonged APD and reduced arrhythmic events in hiPSC-CMs with the *CACNB2*-S480L variant.^59^ In hiPSC-CMs with another variant (p.S142F) in *CACNB2*, both quinidine and bisoprolol reduced arrhythmic events, while carbachol increased them.^60^^,^^61^ These data suggest that *CACNB2* variants can also impact drug effects.

Currently, there is insufficient data regarding pharmacological studies on SQTS with different gene variants. Therefore, further research on the drug effects for SQTS needs to be conducted through in vivo and in vitro experiments. The drugs tested in patients and in SQT-models are summarised in Table 2.

**Table 2:** Summary of tested drugs in short-QT syndrome.

Clinical studies								
Subtypes	Cases	Gene	Mutation	Ionic current affected	Effect on current	Drugs	Main results	References
SQTS1	6	*KCNH2*	N588K	Ikr	↑	Hydroquinidine (+) Flecainide (−) Sotalol (−) Ibutilide (−)	quinidine but not flecainide, sotalol, ibutilide prolonged QTc	^5^
	3	*KCNH2*	N588K	Ikr	↑	Sotalol (−)	The mutation dramatically increased IKr and reduced the affinity of the channel to sotalol.	^34^
	3	*KCNH2*	N588K	Ikr	↑	Quinidine (+)	Quinidine prolonged the QT interval into the normal range and restored the heart rate dependence of the QT interval toward a range of adaptation reported for normal subjects.	^38^
	1	*KCNH2*	N588K	Ikr	↑	Nifekalant (+)	nifekalant prolonged effective refractory period at multiple ventricular sites as well as the QT/QTc interval (from 260/300 to 364/419 ms) on the surface ECG.	^36^
	3	*KCNH2*	T618I	Ikr	↑	Hydroquinidine (+) Sotalol (−)	quinidine but not sotalol prolonged QTc	^37^
	18	*KCNH2*	T618I	Ikr	↑	Quinidine (+) Bepridil (+)	Quinidine with adequate plasma levels was effective in prolonging QTc intervals among 5 cases, but 3 cases still had premature ventricular contraction or non-sustained ventricular tachycardia. Bepridil successfully prevented ventricular fibrillation in 1 case with 19-ms prolongation of the QTc interval.	^16^^,^^21^
SQTS2	1	*KCNQ1*	R259H	Iks	↑	Hydroquinidine (+)	quinidine prolonged QT	^50^
SQTS3	1	*KCNJ2*	D172N	Ik1	↑	Hydroquinidine (+)	quinidine prolonged QT	^50^
SQTS4	1	*CACNA1C*	G490R	ICaL	↓	Hydroquinidine (+)	quinidine prolonged QT	^58^
SQTS5	1	*CACNB2B*	C1422T	ICaL	↓	Hydroquinidine (+)	quinidine prolonged QT	^58^

**Experimental studies**								
Models	Type of SQTS	Gene	Site-directed mutagenesis	Ionic current affected	Effect on current	Drugs	Main results	References
	SQTS3	*KCNJ2*	D172N	Ik1	↑	Chloroquine (+)	Chloroquine but not quinidine terminates IK1-mediated tachyarrhythmias in the mouse heart	^51^
Rabbit	SQTS1					Ranolazine (+) Vernakalant (+)	ranolazine or vernakalant led to a significant suppression of VF	^40^
Guinea pig	SQTS2					TMCC(+)	TMCC reversed pinacidil-and trapidil-induced APD shortening.	^49^
HEK293	SQTS1	*KCNH2*	N588K	Ikr	↑	Quinidine (+) Sotalol (−)	The mutation causes a 20-fold increase in IC50 of d-sotalol but only a 5.8-fold increase in IC50 of quinidine.	^38^
	SQTS1	*KCNH2*	N588K	Ikr	↑	Carvedilol (+)	N588K–*KCNH2* attenuated carvedilol's inhibition of the IKr tail but enhanced carvedilol's IKr end-pulse inhibition.	^39^
	SQTS1	*KCNH2*	T618I	Ikr	↑	Dofetilide (−)	Dofitilide failed to inhibit T618I current.	^21^
	SQTS1	*KCNH2*	T618I	Ikr		Quinidien (+) Sotalol (+)	Quinidine (5 μM) and sotalol (500 μM) had similar inhibitory effects on steady currents measured at +20 mV in WT and T618I but were less effective in inhibiting tail currents of mutant channels.	^16^
	SQTS1	*KCNH2*	I560T	Ikr	↑	Quinidine (+)	The I560T mutation exerted only a modest effect on quinidine sensitivity of IhERG:	^19^
	SQTS2					TMCC(+)	TMCC reduced Iks.	^49^
	SQTS3	*KCNJ2*	D172N	Ik1	↑	Chloroquine (+)	Chloroquine inhibited Ik1 stronger than quinidine	^51^
						Quinidine (+)		
	SQTS3	*KCNJ2*	D172N	Ik1	↑	Styrax (+)	styrax can abolish the inward and outward currents of Kir2.1. The IC50 of styrax for WT, D172N and E299V are similar.	^57^
CHO		*KCNJ2*	E299V	Ik1	↑	Styrax (+)		^57^
	SQTS1	*KCNH2*	N588K	Ikr	↑	Disopyramide (+) Quinidine (+) E−4031 (−)	the N588K-HERG mutation only slightly attenuates I(HERG) blockade by disopyramide (1.5-fold elevation of IC(50)), compared to quinidine (3.5-fold elevation of IC(50)) and E−4031 (11.5-fold elevation of IC(50)).	^35^
	SQTS1	*KCNH2*	N588K/S631A	Ikr	↑	Amiodarone (+) Quinidine (+) Disopyramide (+) E−4031 (−)	The N588K mutation attenuated I (*hERG*) inhibition in the following order: E−4031>amiodarone > quinidine > propafenone > disopyramide.	^4^
	SQTS2	*KCNQ1*	V307L	Iks	↑	Carvedilol (−)	V307L reduced caredilol blockade of Iks.	^39^
	STQS3	*KCNJ2*	D172N	Ik1	↑	Chloroquine (+)	chloroquine inhibited WT Kir2.1 and D172N-Kir2.1 current.	^52^
hiPSC-CMs	SQTS1	*KCNH2*	N588K	Ikr	↑	Ivabradine (+) Ajmaline (+) Mexiletine (+) Flecainide (−) Ranolazine (−) Amiodarone (−)	Ivabradine, mexiletine, and ajmaline but not flecainide, ranolazine, or amiodarone prolonged APD and reduced arrhythmic events in hiPSC-CMs	^6^^,^^7^
	SQTS1	*KCNH2*	N588K	Ikr	↑	Quinidine (+) Disopyramide (+) Sotalol (−)	Application of quinidine and disopyramide, but not sotalol, normalized APD and suppressed arrhythmia induction.	^42^
	SQTS1	*KCNH2*	N588K	Ikr	↑	Quinidine (+) Vernakalant (+) Sotalol (−)	Both AP shortening and arrhythmia irregularities were reversed by quinidine and vernakalant treatment, but not by sotalol.	^43^
	SQTS1	*KCNH2*	N588K	Ikr	↑	Disopyramide (+)	Disopyramide enhanced ICa-L, late INa and INCX, but reduced ISK, leading to APD-prolongation.	^44^
	SQTS1	*KCNH2*	N588K	Ikr	↑	Vernakalant (+)	Vernakalant failed to suppress the hERG channel currents but reduced the Isk and enhanced INCX and prolonged APD.	^45^
	SQTS1	*KCNH2*	N588K	Ikr		Quinidine (+) Sotalol (−) Metoprolol (−)	Quinidine but not sotalol or metoprolol prolonged the action potential duration and abolished arrhythmic activity induced by carbachol.	^41^

**Computational modelling**								
Models	Type of SQTS	Gene	Site-directed mutagenesis	Ionic current affected	Effect on current	Drugs	Main results	References
Computional modelling	SQTS1	*KCNH2*	N588K	Ikr	↑	Quinidine (+) Ivabradine (+) Ajmaline (+) Mexiletine (+)	the modeling detected that quinidine, ivabradine, ajmaline and mexiletine prolonged APD, similar to that detected in hiPSC-CMs.	^46^
	SQTS1	*KCNH2*	N588K	Ikr	↑	Chloroquine (+)	chloroquine prolonged APD and QT interval and declined the T-wave amplitude.	^56^
	SQTS2	*KCNQ1*	V307L V141M	Iks Iks	↑ ↑	Quinidine (+) Quinidine (−)	Quinidine was effective at terminating arrhythmic excitation waves associated with the V307L but not V141M mutation.	^47^
	SQTS2	*KCNQ1*	V307L	Iks	↑	Amiodarone (+)	At the cellular level, amiodarone both at low and high doses prolonged the SQT2 AP duration (APD); at the tissue level, amiodarone at a high dose caused QT prolongation to the physiological range.	^48^
	SQTS3	*KCNJ2*	D172N	Ik1	↑	Amiodarone (+)	Amiodarone increased cellular ERP, prolonged QT interval and decreased the T-wave amplitude.	^54^
	SQTS3	*KCNJ2*	D172N	Ik1	↑	Quinidine (+) Disopyramide (+) E−4031 (+)	At the single cell level, quinidine, disopyramide and E−4031 prolonged APD. Quinidine exhibited significantly better therapeutic effects on SQT3 than disopyramide and E−4031.	^55^
	SQTS3	*KCNJ2*	D172N	Ik1	↑	Chloroquine (+)	chloroquine prolonged APD and QT interval and declined the T-wave amplitude.	^56^

(+), effective; (−), ineffective; ↑, increase; ↓, decrease.

## Long QT syndrome

LQTS, which is defined by the prolongation of the QT interval, was first reported by Jervell and Lange-Nielsen in the 1950s.^62^ It is a genetic cardiac channelopathy. The ACC/AHA/HRS and ESC guidelines clearly defined the diagnostic criteria for LQTS.^10^^,^^63^

### Genotypes of long QT syndrome

So far 17 subtypes of genetic long QT Syndrome (LQTS) have been described (Table 1), with LQTS1, LQTS2, and LQTS3 being the most common subtypes.^64^ In further exploration of the correlation between genes and phenotypes of LQTS, *KCNQ1*, *KCNH2*, and *SCN5A* have been classified as definitive evidence for LQTS1-3, respectively. Three calmodulin genes, *CALM1* (LQTS14), *CALM2* (LQTS15) and *CALM3* (LQTS16), as well as *TRDN* (LQTS17), are classified as definitive/strong evidence subtypes. *CACNA1C* (LQTS8) was classified as moderate evidence. *KCNE1* (LQTS5) and *CAV3* (LQTS9) is classified as limited evidence, while *KCNE2* (LQTS6), *AKAP9* (LQTS11), *ANK2* (LQTS4), *KCNJ5* (LQTS13), *SCN4B* (LQTS10) and *SNTA1* (LQTS12) are classified as disputed evidence for causality in LQTS. *KCNJ2*, which was described as LQTS7 gene, is classified as limited evidence for LQTS, but definitive evidence for Anderson-Tawil syndrome.^64^

LQTS1 is the most common subtype, accounting for 30–35% of individuals with LQTS.^65^ It is caused by the loss-of-function of *KCNQ1* variants.^66^^,^^67^ LQTS2 is the second most common subtype of LQTS and is associated with loss-of-function variants in the *KCNH2* gene.^68^ The characteristic ECG finding of LQTS2 is a notched or bifid T-wave.^69^ Daily life activities such as sudden startle like from loud noise or emotional stress have been identified as triggers for LQTS2 patient.^70^ Compared to patients with LQTS1, LQTS2 patients have a higher risk of life-threatening events.

LQTS3 is caused by gain-of-function mutations of *SCN5A* and accounts for 5–10% of total LQTS cases.^65^^,^^71^ LQTS3 is characterised by a late-onset T-wave,^72^ which is caused by late sodium channel currents and enhancement of phase 2 of the action potential.

Recently, other rare genotypes of LQTS have been discovered,^64^ and they also have a high mortality rate. However, there are very few studies on pharmacologic therapy for these genotypes due to their low incidence.

### Pharmacological studies on the long QT syndrome

Table 3 shows different drugs tested in LQTS patients with different efficacy. This suggests that the impact of gene variants cannot be excluded, although there may be other factors influencing the efficacy of the drug. Most clinical studies focused on the efficacy of β blockers on LQTS, mainly on LQTS1 and LQTS2. Some experimental studies have examined effects of other drugs, such as bupivacaine (a local anaesthetic), on chromanol 293B-induced LQTS1 and E4031-induced LQTS2-like changes in guinea pig cardiomyocytes,^91^ NS1643 (an *hERG* channel activator) on LQTS1 in transgenic rabbits with K_V7.1_ mutants^83^ and ranolazine on dofetilide-induced LQTS2 in canines.^85^ Although these studies observed beneficial effects, they have their limitations, such as drug-induced disease-like changes in animal models, which differ from genetic diseases in humans.

**Table 3:** Tested drugs in long-QT syndrome (LQTS).

Clinical studies								
Subtypes	Cases	Gene	Mutation	Ionic current affected	Effect on current	Drugs	Main results	References
LQTS1	79 69 59	*KCNQ1*		Iks	↓	Propranolol (+) Nadolol (+) Metoprolol (−)	The QTc shortening with propranolol was greater than nadolol and metoprolol. There was a greater risk of BCEs for symptomatic patients initiated on metoprolol compared to propranolol and nadolol.	^73^
	105 20 72 125	*KCNQ1*		Iks	↓	Atenolol (+) Metoprolol (+) Propranolol (+) Nadolol (+)	In LQT1, the risk reduction for first cardiac events was similar among the 4 β-blockers, but in LQT2, nadolol provided the only significant risk reduction (hazard ratio: 0.40 [0.16 to 0.98]).	^74^
	42 10 19	*KCNQ1*		Iks	↓	Bisoprolol (+) Nadolol (+) Atenolol (−)	QTc shortening was observed in individuals on bisoprolol and nadolol but not on atenolol.	^75^
LQTS2	55 32 88	*KCNH2*		Ikr	↓	Propranolol (+) Nadolol (+) Metoprolol (−)	The QTc shortening with propranolol was greater than nadolol and metoprolol. There was a greater risk of BCEs for symptomatic patients initiated on metoprolol compared to propranolol and nadolol.	^73^
	114 46 100 109	*KCNH2*		Ikr	↓	Atenolol (+) Metoprolol (+) Propranolol (+) Nadolol (+)	In LQT1, the risk reduction for first cardiac events was similar among the 4 β-blockers, but in LQT2, nadolol provided the only significant risk reduction (hazard ratio: 0.40 [0.16 to 0.98]).	^74^
	17 6 20	*KCNH2*		Ikr	↓	Bisoprolol (+) Nadolol (+) Atenolol (−)	QTc shortening was observed in individuals on bisoprolol and nadolol but not on atenolol.	^75^
	12	*KCNH2*		Ikr	↓	Mexiletine (+)	After mexiletine, the median QTc decreased by 65 ± 45 ms. In 8 patients (67%), the QTc decreased by ≥ 40 ms with a mean decrease in QTc of 91 ms (P < 0.008). For the 11 patients maintained on mexiletine therapy, there have been no breakthrough cardiac events during follow-up.	^76^
LQTS3	2	*SCN5A*	V411M	INa	↑	Mexiletine (−)	The QTc interval was still longer than 500 ms during follow-up even under oral mexiletine. Case 2 (his aunt) diagnosed as LQT3 suffered from syncope caused by ventricular fibrillation at 35-years-old despite taking mexiletine.	^77^
	34	*SCN5A*	I397F,V411M, L771V,S941N	INa	↑	Mexiletine (+)	Mexiletine significantly shortened QTc (by 63 ± 6 ms; p < 0.0001) and reduced the percentage of patients with arrhythmic events (from 22% to 3%; p = 0.031	^78^
	6	*SCN5A*	DeltaKPQ	INa	↑	Flecainide (+)	Flecainide shortened QT, with an adjusted reduction in QTc of −27.1 ms (95% confidence interval: −36.8 ms to −17.4 ms; P < 0.001). Minimal prolongation in QRS occurred (mean: +2.5 ms), and there were no major adverse cardiac effects.	^79^
	8	*SCN5A*	D1790G	INa	↑	Flecainide (+) Lidocaine (−)	Flecainide shortened QT and also normalized the marked baseline repolarization dispersion in most mutation carriers.	^80^
	5	*SCN5A*	DeltaKPQ	INa	↑	Ranolazine (+)	Ranolazine shortened QTc by 26 ± 3 ms (P < 0.0001) in a concentration-dependent manner. No adverse effects of ranolazine were observed in the study patients.	^79^
LQTS8	1	*CACNA1C*	G402S,G406R	ICaL	↑	Verapamil (+)	Verapamil reduced arrhythmia events and ICD shocks.	^81^
	1	*CACNA1C*	G406R,G402S	ICaL	↑	Mexiletine (+)	QT-interval was shortened on continued anti-arrhythmic treatment with mexiletine, the patient was in stable sinus rhythm and no ventricular arrhythmias occurred.	^82^
	1	*CACNA1C*	G406R	ICaL	↑	Ranolazine (+)	The patient has been completely VT/VF free since ranolazine was added to verapamil.	^82^

**Experimental studies**	**Type of LQTS**	**Gene**	**Site-directed mutagenesis**	**Ionic current affected**	**Effect on current**	**Drugs**	**Main results**	**References**
Models								
Rabbit	LQTS1	*KCNQ1*	Y315S Y315S	Iks	↓	NS1643 (+) Docosahexaenac acid (−)	In vivo, NS1643 shortened the QTc significantly in LQT1 compared with vehicle. In Langendorff experiments, NS1643 significantly shortened the APD (90) in LQT1 and control rabbits. Docosahexaenoic acid significantly shortened QTc in vivo in WT and LQT2 but not in LQT1 and LQT5 rabbits.	^83^^,^^84^
	LQTS2	*KCNH2*	G628S	Ikr	↓	Docosahexaenac acid (+)		
	LQTS5	*KENE1*	G52R	Iks	↓	Docosahexaenac acid (−)		
Canine	LQTS2			Ikr	↓	Ranolazine (+)	ranolazine reduced the number of TdP episodes from 10 ± 3 to 3 ± 1 (p < 0.05) and partially reversed the increase of beat-to-beat variability of repolarization with no abbreviation of the dofetilide-induced QT prolongation.	^85^
HEK293	LQTS3	*SCN5A*	DeltaKPQ	INa	↑	Flecainide (+)	Compared with WT, DeltaKPQ I(Na) was more sensitive to flecainide, and flecainide preferentially inhibited late I(Na) compared with peak I(Na)	^86^
	LQTS3	*SCN5A*	Q1475P			Mexiletine (+) Phenytoin (+)	Mexiletine and phenytoin similarly rescued some of the gating defects. Chronic incubation with mexiletine, but not phenytoin, rescued the Nav1.5-Q1475P trafficking defect, thus increasing mutant channel expression.	^87^
hiPSC-CMs	LQTS2	*KCNH2*	A614V	Ikr	↓	Nifedipine (+) Pinacidil (+) Ranolazine (+)	Pinacidil and nifedipine shortened APD, abolished EAD and triggered arrhythmias. Ranolazine did not prolong APD but showed antiarrhythmic effects.	^88^
	LQTS2	*KCNH2*	G1681A	Ikr	↓	Nicorandil (+) PD118057 (+) Propranolol (+) Nadolol (+)	Nicorandil and PD118057 shortened APD and in some cases could abolish EADs. Propranolol and nadolol reversed isoprenaline induced EADs.	^89^
	LQTS3	*SCN5A*	R1644H	INa	↑	Mexiletine (+) Ranolazine (+) Phenytoin (+)	Mexiletine, ranolazine and phenytoin shorten APD and field potential durations specifically in LQT3 hiPSC-CMs and antagonized EADs in a dose-dependent manner.	^90^
Computational modelling	LQTS1	*KCNQ1*		Iks	↓	Bupivacaine (+)	the incidence of AP prolongations in the presence of 3 μmol/L bupivacaine was significantly increased from 6% in control myocytes to 24% in LQT1-like but not in LQT2-like myocytes. Computational modeling supported the concept that this bupivacaine-induced the AP prolongations in the control and LQT1-like myocytes were caused by inhibition of hERG channels.	^91^
	LQTS2	*KCNH2*		Ikr	↓	Bupivacaine (+)		

(+), effective; (−), ineffective; ↑, increase; ↓, decrease.

Experimental studies that focus on the effects of drugs on specific gene variants can provide valuable information about how these variants influence the efficacy of the drugs. For instance, there have been studies examining the effects of siRNA on *hERG*-E637K in HEK cells,^92^ ranolazine on the *KCNH2*-A614V variant in hiPSC-CMs from a patient with LQTS2,^88^ and propranolol, nadolol and potassium channel activators (nicorandil and PD118057) on *KCNH2*-A561V.^89^ Positive effects were observed in these studies, but it is important to note that the effects of these drugs on other variants were not explored.

In a recent study, the genotype-specific benefits of docosahexaenoic acid (DHA) were demonstrated in rabbits carrying the *KCNH2*-G628S variant, but not in those carrying the *KCNQ1*-Y315S or *KCNE1*-G52R variants, or a combination of *KCNH2*-G628S + and *KCNE1*-G52R (79). This indicates that the effects of DHA are dependant on the specific variant present.

Given that LQTS3 is caused by a gain of function in the *SCN5A* channel, Na^+^ channel blockers have the potential to be effective treatment options. Schwartz and Mazzanti conducted a study and found that mexiletine not only shortened QTc, but also reduced life-threatening arrhythmic events in LQTS3 patients with *SCN5A* gene variants, including I397F, V411M, L771V, S941N, P1332L, DelQKP1507–1509, R1623Q, R1626P, R1644H, E1784K, and M1652R.^76^, ^77^, ^78^ They also successfully generated LQTS3-hiPSC-CMs with the *SCN5A*-R1644H variant and proved that mexiletine effectively in shortened APD and reduced early-after-depolarisation (EADs).^90^ Therefore, mexiletine may be a promising therapeutic agent for LQTS3. Additionally, it was discovered that flecainide shortened QTc in LQTS3 patients with the *SCN5A*-ΔKPQ deletion^93^ or *SCN5A*-D1790G.^80^ In vitro studies showed that flecainide has a high affinity for the Nav1.5 channel protein and can improve impaired inactivation caused by the ΔKPQ deletion in HEK293 cells.^86^ Furthermore, ranolazine,^79^^,^^94^ phenytoin,^87^ verapamil^81^ and mexiletine^82^ have also shown beneficial effects in LQTS3 or LQTS8.

Recently, studies have demonstrated that SGK1 inhibitors can shorten APD in different scenarios. For example, they have been shown to be effective in iPSC-CMs with the *SCN5A*-N406K variant^95^ and in dofetilide-treated iPSC-CMs.^96^ Additionally, SGK1 inhibitors have been found to be effective in cells with LQTS2 variants (KCNH2-p.A561V/p.A614V/p.G628S/IVS9-28 A/G) and LQTS1 variants (*KCNQ1*-p.R594Q and *KCNQ1*-p.A341V) but not in cells with the LQTS1 *KCNQ1*-p.A341V or *KCNQ1*-p.Y315S variant.^97^ These findings suggest that SGK1 inhibitors may be potential candidate drugs for treating LQTS, particularly LQTS3, as they can selectively reduce *I*_Na-L_.^95^

Furthermore, the effects of SGLT2 inhibitor on *I*_Na-L_ and APD/QT have been described. Empagliflozin, an SGLT2 inhibitor, was shown to reduce *I*_Na-L_ in cardiomyocytes from mice with heart failure and in cells carrying *SCN5A*-R1623Q or *SCN5A*-ΔKPQ.^98^ It also attenuated sotalol-induced QT prolongation^99^ or amitriptyline-induced QT prolongation in rats.^100^ In patients treated with SGLT2 inhibitors as an add-on therapy to metformin, the QT interval was found to be shorter compared to patients treated with other glucose-lowering agents.^101^ These findings support the potential use of SGLT2 inhibitors for the treatment of LQTS3.

While there is limited data on the effects of SGLT2 and SGK1 inhibitors in LQTS patients or models, the study exploring the therapeutic effects of those inhibitors on LQTS may offer a new pharmacotherapy option. Further research in this area is needed to establish the clinical significance of SGLT2 or SGK1 inhibitors in LQTS therapy.

There is limited research on the pharmacological therapy of other rare genotypes of LQTS. The drugs tested in LQTS-patients and models are summarised in Table 3.

## Mechanisms underlying the impact of gene variants on drug effects

The aforementioned clinical and experimental studies have shown that drug effects can vary among patients with different gene variants.

There are two basic processes, namely pharmacokinetic and pharmacodynamic variation, that can contribute to the variability of drug effects. These processes can be influenced by both genetic and non-genetic factors. This review will primarily discuss the variability of drug effects in relation to ion channel gene variants.

### Gene variants can change channel gating and thereby drug effects

Multiple variants have been identified that interrupt the fast inactivation process of the *hERG* channel, resulting in reduced effects of drugs e.g. methanesulfonanilides.^102^^,^^103^ One such variant is the *KCNH2*–N588K missense variant, which is located in the linker between the S5 and S6 segments of the *hERG* channel. This variant can impair channel inactivation and decrease the channel's affinity for class III antiarrhythmic drugs.

Regarding the mechanism of how channel inactivation influences drug effects, it has been suspected that when a drug like sotalol binds to the channel in its open state,^104^ the inactivation of the channel can stabilise the drug on its binding site, thereby increasing the drug's affinity. As a result, the inactivation-impairing variant N588K is resistant to sotalol because the defect in inactivation reduces the drug's affinity. This may help us understand why *KCNH2*–N588K is resistant to class III antiarrhythmic drugs, which depend on inactivation.

Besides, it has been suggested that the fast inactivation process of the *hERG* channel can cause allosteric changes in the inner mouth region. This in turn facilitates the development of a high affinity binding site for certain drugs.^104^^,^^105^ Several hERG variants, such as N588K, S620T,^103^ T623A, S624A, V625A, G648A, Y652A, F656A,^106^ G628C/S631C^104^ and S631A,^107^^,^^108^ have been tested to determine the relationship between drug potency and inactivation. These amino-acid residues associated with inactivation are located in different regions near or at the extracellular face of the channel: 1) the turret, 2) the domain of the outer mouth of the pore (C-terminal side of the pore loop), and 3) inside the pore loop. It is known that most drugs that block the hERG channel with high affinity access the channel pore cavity from the intracellular side when the channel is open, and the canonical high-affinity drug-binding site largely depends on two aromatic residues (F656 and Y652).^106^ However, it is unclear how the inactivation, which is dependant on residues near the extracellular face of the channel, affects drug effects occurring in residues in S6 near the intracellular side of the channel pore. The phenomenon that the effect of disopyramide is only slightly reduced by either the N588K or S631A single variant, but significantly attenuated by the double variants, suggests that *hERG* channel inactivation may involve two synergistic processes, each independently influenced by variants at different sites such as the turret or outer mouth of the pore.

Sodium channel blockers such as mexiletine, flecainide, and ranolazine are potential drugs for treating LQT3 because they can selectively inhibit the late sodium channel current. However, the response of LQT3 patients to these drugs can vary, indicating that the effects of the drugs may depend on genetic variants.

Experimental data (Table 4) on the impact of LQTS3 variants show that different variants can affect sodium channel gating kinetics in different or similar ways.^110^, ^111^, ^112^, ^113^, ^114^, ^115^, ^116^, ^117^, ^118^, ^119^, ^120^, ^121^, ^122^ Additionally, a variant can respond differently or similarly to different drugs. These findings suggest that relying solely on cellular electrophysiological data, such as activation, inactivation and recovery from inactivation, is insufficient to determine specific efficacy of different drugs for different gene variants or to guide personalised therapy.

**Table 4:** The impact of long-QT syndrome 3 (LQTS3) variants on sodium channel gating and drug effects.

Variant in *SCN5A*	INa–P	INa-L	Act	Inac	Rec	Drug	Ref
I397F		Ic	LS	RS	0	Mex (+)	^109^
V411M		Ic	LS	0	Dc	Mex (+)	^109^
L711V		0	LS	RS	0	Mex (−)	^109^
S941N		Ic	LS	RS	Dc	Mex (−)	^110^
		Ic	0	LS	Ac	Mex (−)	^109^
P1332L		Ds	LS	0	Dc	Mex (+)	^110^
		Ic	0	LS	Ac	Mex (+)	^109^
deltaKPQ		Ic	RS	LS	Dc	Mex (+)	^109^
						Fle (+)	^86^
				LS	Dc	Fle (+)	^111^
							^109^
R1623Q		0	RS	LS	0	Mex (+)	^109^
	Ds	Ic	RS	RS			^112^
R1626P		Ic	RS	LS	0	Mex (+)	^109^
		Ic	0	LS	0	Mex (+)	^110^
R1644H		Ic	RS	LS	Dc	Mex (+)	^109^
L1650F		0	RS	0	Ac	Mex (+)	^109^
M1652R		0	RS	RS	Ac	Mex (−)	^109^
		Ic	0	RS	Dc	Mex (−)	^110^
I1771M		0	RS	LS	0	Mex (−)	^109^
E1784K		Ic	RS	LS	Ac	Mex (+)	^109^
N1352S	Ds	Ic	LS	RS	0	Mex (+)	^113^
Y1767C	Ds	Ic	LS	0	Ac	Ran(+)	^114^
	Ds	Ic	0	RS		Mex (−)	^115^
A1656D	Ds	Ic	RS	LS		Mex (+) Ran(−) Fle (−)	^116^ ^116^ ^116^
V1763M	Ds	Ic	RS	RS	Ac	Mex (+)	^117^
L1786Q	Ds	Ic	RS	LS		Fle (+)	^118^
1795insD				LS	Ac	Fle (+)	^111^
I1756C					Ac	Fle (+)	^119^
N406K	Ds	Ic	LS	RS	0	Mex (+)	^120^
F1473C		Ic	0	LS	Ac	Mex (+)	^121^
		Ic	0	LS	Ac	Mex (+) Fle (+) Ran(+)	^122^ ^122^ ^122^
					
					

Ds, decrease; Ic, increase; RS, rightward shift; LS, leftward shift; 0, no change; Dc, decelerate; Ac, acelerate; (+), effective; (−), no effect; Pl, prolongation, INa-p, peak INa; INa-L, late INa; Act, activation; Inac, inactivation; Rec, recovery; Ref, reference.

Zhu et al. conducted a study to investigate how certain *SCN5A* variants differentially impacted mexiletine effects.^109^ Their findings revealed that mexiletine causes changes in the conformation of the voltage-sensing domain (VSD) in Domain-III (DIII) of the cardiac Na^+^-channel. The DIII-VSD was found to be influenced by various LQTS3 variants, indicating that changes in the DIII-VSD conformation can impact the blocking effect of mexiletine.

### Position and placed amino acid of variants impact drug effects

The C-type inactivation-deficient variants, S620T and S631A in the *hERG* channel, reduce verapamil blockage. This suggests that inactivation is crucial for verapamil blockade. Interestingly, the *KCNH2*-S620T variant decreases verapamil-induced blockage 20 times more than the *KCNH2*-S631A variant does. It has been suggested that verapamil enters the cell membrane as a neutral form and acts at a site within the pore from the intracellular side, which likely contains the serine at position 620.^107^ Furthermore, it was observed that *KCNH2*-S620C, *KCNH2*-S620T, *KCNH2*-S631A and *KCNH2*-S631V significantly increase the half maximum inhibitory concentration (IC50) of dofetilide for blocking the channel. Notably, *KCNH2*-S620T and *KCNH2*-S631V have a much stronger impact than *KCNH2*-S620C and *KCNH2*-S631A, respectively.^103^ These findings indicate that both the variant site and the replaced amino acid are important factors affecting the impact on drug effects.

Mikuni et al. discovered that both the F340A and F340C mutants of *KCNQ1* reduced the effect of isoflurane. However, *KCNQ1*-F340A was found to be more effective than *KCNQ1*-F340C in reducing the isoflurane effect.^123^ This difference may be attributed to the fact that phenylalanine and alanine are non-polar, while cysteine has an uncharged polar character.

### Variants impact drug effects via changing regulation of channel function

Beside gene variants, there are other factors that can affect the effects of drugs. Abdelsayed et al. demonstrated the combined impact of external triggers and an *SCN5A* variant on the effects of ranolazine.^124^ They discovered that both high temperature (41 °C) and high intracellular calcium weakened the effectiveness of ranolazine in reducing *SCN5A*-E1784K late I_Na_. The fast inactivation kinetics of E1784K were not influenced by temperature, indicating that channel inactivation likely does not contribute to the sensitivity of late I_Na_ to temperature. Instead, a structural rearrangement can occur in DIVS4 at high temperature, causing the voltage sensor to a state that can enhance the conductance in E1784K.^125^ As a result, the blockade of ranolazine is reduced. It is speculated that the *SCN5A*-E1784K mutation can disrupt either the calcium-dependant or calcium–independent interactions between the DIII-DIV linker and the C-terminal domain resulting in the inhibition of the calcium-dependant facilitation in Nav1.5. This mutation may create a high entropy, unstable structure in the C-terminal domain. When the intracellular calcium level rises, the calcified calmodulin can reduce its affinity for the IQ motif, thereby increasing the entropy of the C-terminal domain.^126^ The increased entropy caused by calcium-bound calmodulin in the C-terminal domain appears to hinder ranolazine from reaching the inner vestibule of the channel pore.

Galleano investigated the mechanism behind the disruption of channel steady-state inactivation caused by phosphorylation of *SCN5A*-Y1495.^127^ Surprisingly, they discovered that the *SCN5A*-Q1476R mutant did not independently affect channel inactivation, but it enhanced the impairment of steady-state inactivation resulting from phosphorylation at the Y1495 site by promoting the release of the inactivation particle. These findings emphasise that gene variants can greatly strengthen the functional consequences including drug effects of *SCN5A* phosphorylation. This may have implications for understanding mutational phenotypes and guiding future drug development. Furthermore, it was observed that the PKC activator OAG strongly inhibited the late I_Na_ of the mutant (Y1795C, Y1795H, and DeltaKPQ) but not the WT channels.^128^ The effect of OAG on late I_Na_ was reduced by the PKC inhibitor staurosporine, eliminated by the S1503A variant, and replicated by the S1503D variant. This suggests that S1503 is the PKC phosphorylation site in *SCN5A*. An interesting finding in this study is that certain non-PKC sites (Y1795C, Y1795H, and DeltaKPQ) rendered the *SCN5A* channel sensitive to PKC, indicating an interaction between different sites in the channel.

### Variants impact drug effects by altering channel trafficking

Ruan et al.^129^ reported that the *SCN5A*-F1473S variant expressed in HEK293 cells exhibited a significantly inhibited peak I_Na_. Immunostaining revealed that the mutant *SCN5A* channel remained in the cytoplasm, indicating a trafficking defect. However, the use of mexiletine was able to rescue the trafficking defect of F1473S, leading to an increase in the peak I_Na_. Another study conducted by Zhang found that alpha-allocryptopine significantly enhanced the peak I_Na_ of the *SCN5A*-T353I variant, but not the WT- *SCN5A* channels.^130^ *SCN5A*-T353I is a mutant with impaired trafficking, but alpha-allocryptopine was found to increase the trafficking of this mutant to plasma membrane, resulting in an increase in the peak I_Na_. This finding helps explain why *SCN5A*-T353I is more susceptible to the effect of alpha-allocryptopine and suggests that variants that affect channel trafficking may have a particularly significant impact on the effects of drugs that also influence channel trafficking.

### Variants impact drug effects through associated subunits

A study revealed that ML277 and R-L3 enhanced Kv7.1 in homozygote *KCNQ1-KCNE1*-G52R and *KCNQ1-KCNE1*-L51H, as well as in channel complexes that imitate heterogeneous expression of Kv7.1 variants found in patients. However, these drugs did not have the same effect on wild type channels. Similar results were observed for the *KCNQ1*- S546L/*KCNE1* variant when exposed to ML277 and R-L3. Both drugs could affect *KCNQ1* and *KCNE1* variants, but ML277 was more effective than R-L3. Interestingly, the *KCNE1*-D76N variant had the greatest impact on the effects of both drugs.^131^ It was discovered that *KCNE1* subunits can interact with the putative binding site for R-L3 in the S6 domain of the *KCNQ1* subunit, which is facing away from the central cavity.^132^ Additionally, previous data indicated that *KCNQ1* was more sensitive to R-L3 compared to the *KCNQ1* plus *KCNE1* channel.^133^ This suggests that R-L3 and the *KCNE1* subunit may compete for the same binding site on the *KCNQ1* subunit, thereby influencing the effects of R-13 on the *KCNQ1* channel. These data suggest that the ancillary subunit is important not only for *KCNQ1* channel function but also for drug effects on the channel.

## Summary and conclusion

The availability of drug tests for in SQTS patients is currently limited. So far, only quinidine has been proven effective in SQTS patients who have *hERG* variants, but this has been confirmed only through small studies conducted at individual centres. Quinidine has also been tested in SQTS patients with *KCNQ1*, *KCNJ2*, *CACNA1C* and *CACNB2* variants, but the number of patients included in these studies is too small to determine its efficacy.

Currently, β-blockers are the preferred first-line drugs for the pharmacological treatment of LQTS. Mexiletine, flecainide, and ranolazine may be considered as alternative drugs for LQTS3. However, the response of LQTS to β-blockers and sodium channel blockers can vary depending on the genotype. There is not enough available data on drug testing in patients with other types of LQTS to draw meaningful conclusions.

Experimental studies on drug effects have tested numerous drugs using different models including animal models, computer models, heterologous expression systems and hiPSC-CMs with various gene variants. Data from these studies have shown that gene variants in hERG, *KCNQ1* and *SCN5A* not only alter ion channel function, but also affect how drugs work.

Ion channel variants can influence channel conformation, gating, drug binding sites, trafficking and sensitivity to intracellular regulators, all of which can impact the channel's response to drugs. Therefore, it can be concluded that the effects of drugs may vary in SQTS and LQTS patients with different variants. Conducting variant-specific drug testing may be helpful in identifying suitable drugs for SQTS and LQTS, particularly for genotype-guided therapy of these diseases (Fig. 1).

## Outstanding questions

The cardiac channelopathies, SQTS and LQTS, are genetic diseases associated with malignant arrhythmias. However, the severity of risk can vary among different genotypes. Optimal therapy for these conditions is still lacking. In the case of LQTS, beta-blockers and sodium channel blockers can be used. For SQTS, only a limited number of effective drugs have been identified in limited studies, with quinidine being recommended for clinical application. Experimental studies have investigated more drugs in different models with different gene variants, providing helpful information. However, many questions still remain based on the available data. For example, the currently effective drugs have only been tested on patients with certain gene variants, and their effectiveness for other untested variants is unknown. Some drugs have only been tested in experimental models, and whether they are effective in patients is uncertain. Additionally, only a limited number of drugs have been investigated for most of the detected pathogenetic variants, and it remains to be seen if other untested drugs can be effective for these variants. The impact of variants on drug effects may also be synergistic, but data on this are very limited. Further examination is needed to determine if double or multiple variants differentially affect drug effects. Future studies will benefit from new techniques such as hiPSC plus gene editing, computer modelling, biological information and artificial intelligence techniques. These advancements have the potential to promote the design, development and testing of drugs for cardiac channelopathy with specific gene variants. It is anticipated that more effective drugs for genotype-specific therapy of SQTS and LQTS will be discovered in the near future. On the other hand, the application of gene-editing or gene-therapy in patients may reduce or even eliminate the need for the development of genotype-specific therapies by correcting pathogenic gene variants.

## Discrepancies and limitations of the current studies

When it comes to clinical studies on SQTS, the number of published cases is limited. This is related to the absence of a large international registry including enough patients. Therefore, the published data of small single centre studies included very few cases. At maximum, e.g. for hydroquinidine, 16 patients have been reported showing a reduction of arrhythmic events to 0%, but nevertheless no information in these cases on the underlying gene variant is available.^50^ At cellular level two studies showed a possible efficacy of hydroquinidine regarding prolongation of action potential duration and anti-arrhythmic effect in SQTS carrying T618I. However, the clinical data of a small study of SQTS carrying T618I are not conclusive.^21^ These data show a prolongation of the QTc interval but not an anti-arrhythmic effect in all cases. A further important limitation in SQTS is that different genes are related to SQTS and there is an unmet need to model these in animal models and ideally in addition in hiPSC-CMs. Another important limitation is that in up to 70% of SQTS cases the genetic cause is not elusive yet, which is important to understand the drug–gene interaction in SQTS.

When it comes to LQTS, obviously the same problem is common. e.g. non-selective beta-blockers are recommended in patients according to current guidelines. However, clinical studies are not comprehensive and conclusive regarding this point. Whereas in large number of LQTS patients nadolol seems to be more effective compared to propranolol, other studies including smaller number of patients showed that nadolol might be less effective than propranolol.^73^^,^^134^ Besides the patient number, another limitation of most clinical studies is that the impact of gene variants on drug effects, which may be a reason for the discrepancy of clinical data on drug efficacy in different patients, was not investigated.

Of note, regarding the clinical studies in LQTS and SQTS the current knowledge is based on registries rather than randomised clinical studies. Therefore, randomised clinical studies and/or at least prospective data evaluation according to a standard protocol is required to answer important questions and to understand these discrepancies of current literatures.

## Contributors

ZZ, XZ, YZ, IA and IE contributed to the conception and design of the review. The first draft was written by ZZ, XZ, KN, WS, WX and NH, AM, AW, ZZ, XZ, YZ, IA and IE revised the manuscript. All authors approved the submitted version.

## Declaration of interests

All authors have no conflicts of interest to declare.
